# Inflammatory Monocytes Are Rapidly Recruited to the Post-Ischaemic Liver in Patients Undergoing Liver Transplantation and Cytokines Associated with Their Activation Correlate with Graft Outcomes

**DOI:** 10.3390/cimb47010049

**Published:** 2025-01-14

**Authors:** Francis P. Robertson, Antonia O. Cuff, Victoria Male, Graham P. Wright, Laura J. Pallett, Barry J. Fuller, Brian R. Davidson

**Affiliations:** 1 Division of Interventional and Surgical Science, Royal Free Campus, University College London, London NW3 2QG, UK; b.fuller@ucl.ac.uk (B.J.F.); b.davidson@ucl.ac.uk (B.R.D.); 2 Department of Surgery, School of Medicine, Gilmorehill Campus, University of Glasgow Medical School, Glasgow G12 8QQ, UK; 3 Division of Biomedical Sciences, Warwick Medical School, Gibbet Hill Campus, University of Warwick, Coventry CV4 7AL, UK; antonia.cuff@warwick.ac.uk; 4 Department of Metabolism, Digestion and Reproduction, Chelsea and Westminster Hospital Campus, Imperial College London, London W12 0NN, UK; v.male@imperial.ac.uk; 5 School of Applied Science, Edinburgh Napier University, Edinburgh EH11 4BN, UK; g.wright2@napier.ac.uk; 6 Institute of Immunity and Transplantation, Division of Infection and Immunity, University College London, London NW3 2PP, UK; laura.pallett@ucl.ac.uk; 7 Department of HPB and Liver Transplant Surgery, Royal Free Hospital, London NW3 2QG, UK

**Keywords:** liver transplantation, ischaemia–reperfusion injury, CD4+ T cells, inflammatory monocytes

## Abstract

Liver ischaemia–reperfusion (IR) injury remains a major cause of morbidity and mortality following liver transplantation and resection. CD4+ T cells have been shown to play a key role in murine models; however, there is currently a lack of data that support their role in human patients. *Methods:* Data on clinical outcomes and complications were documented prospectively in 28 patients undergoing first elective liver transplant surgery. Peripheral blood samples were collected at baseline (pre-op), 2 h post graft reperfusion, immediately post-op, and 24 h post-op. A post-reperfusion biopsy was analysed in all patients, and in five patients, a donor liver biopsy was available pre-implantation. Circulating cytokines were measured, and T cells were analysed for activation markers and cytokine production. *Results:* Circulating levels of cytokines associated with innate immune cell recruitment and activation were significantly elevated in the peri-transplant period. High circulating IL-10 levels corresponded with the development of graft-specific complications. The proportion of CD4+ T cells in the peripheral circulation fell throughout the peri-operative period, suggesting CD4+ T cell recruitment to the graft. Although TNFα was the predominant cytokine produced by CD4+ T cells in the intrahepatic environment, the production of IFNγ was significantly upregulated by circulating CD4+ T cells. Furthermore, we demonstrated clear recruitment of inflammatory monocytes in the peri-operative period. In donor-and-recipient pairs with a mismatch at the HLA-A2 or A3 allele, we demonstrated that inflammatory monocytes in the liver are recipient-derived. *Discussion:* This is the first study to our knowledge that tracks early immune cell responses in humans undergoing liver transplantation. The recruitment of inflammatory monocytes from the recipient and their cytokine release is associated with liver-specific complications. Inflammatory monocytes would be an attractive target to ameliorate ischaemia–reperfusion injury.

## 1. Introduction

Liver transplantation is the treatment of choice for patients with both acute and chronic end-stage liver failure. As outcomes following liver transplantation have improved, the indications for liver transplantation have widened and a shortage of suitable organ donors has developed. This has resulted in the increased use of grafts from marginal donors such as the elderly, those with a steatotic liver, and those from donors following cardiac death (DCD). The use of liver DCD grafts in the UK has increased from 6.9% in 2005 [1] to 28.7% in 2023 [2]. DCD grafts suffer a more prolonged ischaemic injury during the retrieval stage. The use of grafts from DCD donors is associated with reduced patient and graft survival [1,3].

Ischaemia–reperfusion (IR) injury is the damage that occurs to an organ when its blood supply is interrupted and reconstituted. It is a major cause of morbidity and mortality following liver transplantation and is believed to account for up to 10% of early graft loss [4].

Many molecular pathways have been identified that drive IR injury, but the immune system has been shown to play a major role [5]. Athymic mice, which lack T cells, are protected from the reperfusion phase of IR injury despite suffering the same ischaemic damage [6]. The reconstitution of CD4+ T cells makes these mice susceptible to IR injury, and this has been replicated in several studies [7,8]. The same phenomenon is not seen when CD8+ T cells are reconstituted. This has also been shown in renal [9], intestinal [10], and pulmonary [11] IR injury. The rapid speed of this CD4+T cell response is atypical, and studies have shown that it is not the antigen-dependent activation of T cells that drives it, as blocking the antigen activation of T cells with MHC Class II antibodies has no effect on IR injury [12,13]. There may be a role for inflammatory monocytes with activation by Damage-Associated Molecular Patterns (DAMPs) [14]. Monocytes are rapidly recruited (within hours) [15] to damaged or ischaemic tissue by CCL2 [16]. Furthermore, there is evidence of an interplay between the innate immune cells and CD4+ T cells [13]. TLR4 has been highlighted as a key pathway in immune activation, both in CD4+ T cells and in the closely associated dendritic cells [13], and this would be in keeping with the timescale of IR injury. When linked with further studies that have identified HMGB1, which interacts with TLR4, as an essential mediator of IR injury [17,18] it is more likely that the TLR pathway of immune activation, rather than TCR stimulation, is responsible for propagating IR injury.

Although there is extensive evidence of immune cell infiltration and activation in murine models of IR injury, there is very little evidence in humans.

An analysis of cytokines before and after human liver transplant demonstrated that higher levels of CCL2, IL8, CCL5, CXCL9, IP10, and IL-2 were associated with the development of early allograft dysfunction (EAD), which is associated with graft preservation and IR injury [19]. However, this study was limited to circulating cytokine analysis in 73 patients, and samples were only collected pre-operatively and on days 1, 7, 14, and 30 post-op. No liver biopsies were available for analysis.

A study on human living donor liver transplantation measured cytokine levels pre-operatively and on the 7th day post-operatively in 226 patients. It found that higher post-operative serum levels of IL-6 and IL-17 were associated with EAD, but these were only measured on post-operative day 7 [20]. We know that graft injury occurs early following liver transplant, and identifying early immune changes at this stage will lead to therapies that ameliorate IR injury.

Thus, the aim of this study was to map peripheral and liver graft immune cell changes and their associated cytokines in the early peri-operative period and correlate these findings with graft and patient outcomes.

## 2. Methods

### 2.1. Patient Samples, Plasma, and Immune Cell Isolation

Blood, liver, and perfusate samples were collected from adult patients undergoing first elective deceased donor liver transplantation at the Royal Free Hospital, London, who were recruited to the Remote Ischaemic Preconditioning in Orthotopic Liver Transplantation (RIPCOLT) study (clinical trial number 8191; ethical approval number 11/H0720/4) [21,22].

A total of 50 mls of peripheral arterial blood were collected from the routinely placed arterial line at the following time intervals:

- Baseline (following the induction of anaesthesia but before abdominal incision);
- Immediately before removal of the recipient’s liver;
- Two hours post-reperfusion in the recipient of the donor graft;
- Twenty-four hours post-operatively.

The University of Wisconsin solution that the grafts were transported in and the human albumin solution that was used to flush the grafts prior to reperfusion were collected. A post-reperfusion biopsy was performed routinely. Following an ethical amendment, a pre-reperfusion biopsy was also performed on 5 patients.

A total of 10 mls of the arterial blood sample was immediately centrifuged at 1000× *g* for ten minutes to obtain plasma, which was stored at −80 °C until analysis.

### 2.2. Isolation of Immune Cells

To accurately reflect the in vivo environment, immune cells were isolated and analysed fresh from liver biopsies, blood, and transport fluid (without further stimulation) through manual homogenisation and density centrifugation (see Supplementary Materials, experimental methods) as previously described [23]. Peripheral blood mononuclear cells and intrahepatic lymphocytes were similarly stained to allow analysis by flow cytometry.

### 2.3. Plasma Cytokine Measurements

Plasma concentrations of IL-2, IL-6, IL-10, IFN-γ, and TNF-α were measured using a LEGENDplex multiparametric bead-based immunoassay (Human Th cytokine 5 plex mix and match subpanel Biolegend, London, UK). The LEGENDplex was performed according to the manufacturer’s guidelines.

Plasma concentrations of CCL2 (Biolegend, London, UK), CCL5 (Biolegend, London, UK), IL-8 (Biolegend, London, UK), and IL-17A (Biolegend, London, UK) were measured by ELISA according to the manufacturer’s guidelines.

### 2.4. Flowcytometry

For cell surface markers, cells were incubated with fluorescently conjugated antibodies at 4 °C for 30 min. For intracellular staining, cells were fixed and permeabilised with Human FoxP3 Buffer Set (BD Bioscience, Wokingham, UK). Dead cells were identified with a fixable viability dye (ThermoFisher, Horsham, UK) and excluded. Samples were analysed on an LSRII flow cytometer running FACSDiva software v9.0 (BD Bioscience, Wokingham, UK). The list of antibodies used and the gating strategy are contained in Supplementary Materials, experimental methods.

### 2.5. Data Analysis and Statistics

All flow cytometry data were analysed using FlowJo V.9-10 (FlowJo LLC). Statistical analysis was carried out on Prism V.10 (GraphPad). Normality of the data was assessed using the Shapiro–Wilk test. Median average values and nonparametric testing was used throughout.

## 3. Results

Samples were collected from 28 patients undergoing elective first liver transplant surgery. Background clinical information and peri-transplant outcomes are shown in Table 1.

### 3.1. Cytokines Associated with Inflammatory Monocyte Recruitment and Activation Were Elevated in the Peri-Transplant Period and Correlated with Graft Outcomes

Circulating levels of Chemokine Ligand 2 (CCL2), Chemokine Ligand 5 (CCL5), Interleukin 6 (IL-6), Interleukin 8 (IL-8), Interleukin 10 (IL-10), and Interleukin 17A (IL-17A) were raised significantly from baseline following the mobilisation of the recipient liver and at 2 h post-reperfusion of the donor graft. Circulating levels of these cytokines returned to pre-operative levels within 24 h following the transplant surgery (Table 2). Circulating levels of Interleukin 2 (IL-2), Interferon Gamma (IFNγ), and Tumour Necrosis Factor Alpha (TNFα) were not significantly raised at any timepoint during the transplant procedure or in the first 24 post-operative hours.

Fifteen patients suffered from early allograft dysfunction (EAD) [24]. These patients had significantly higher plasma levels of IL-10 at 2 h post-reperfusion than those who did not suffer from EAD [747.69 (462.15–1336.7) pg/mL vs. 420.43 (338.05–561.39) pg/mL, *p* = 0.008]. Patients who suffered from EAD had significantly higher levels of CCL2, the inflammatory chemokine, which attracts monocytes, at 24 h post-reperfusion [105.96 (70.61–238.45) pg/mL vs. 53.49 (35.77–77.73) pg/mL, *p* = 0.013]

There were no correlations between circulating cytokine levels post-reperfusion and ischaemic liver injury as measured by liver aspartate transferase levels on day 3 [25]. However, elevated CCL2 levels, measured at 24 h post-reperfusion significantly correlated with increased AST levels on day 3 [*p* = 0.009], a surrogate marker for graft and patient outcome [25].

Six patients developed a graft-specific complication within 3 months of liver transplant (portal vein thrombosis, hepatic artery thrombosis, bile leak, and biliary stricture). These patients had significantly higher circulating levels of IL-10 at 2 h post-reperfusion [1507.22 (951.87–343.72) pg/mL vs. 462.15 (343.72–718.3) pg/mL, *p* = 0.006] but significantly lower IL-10 levels at 24 h post-operation [4.39 (3.34–5.1) pg/mL vs. 10.10 (4.92–38.23) pg/mL, *p* = 0.027].

### 3.2. The Percentage of CD4+ T Cells in the Peripheral Circulation Falls Post-Reperfusion of the Liver Graft

**Figure 1:**
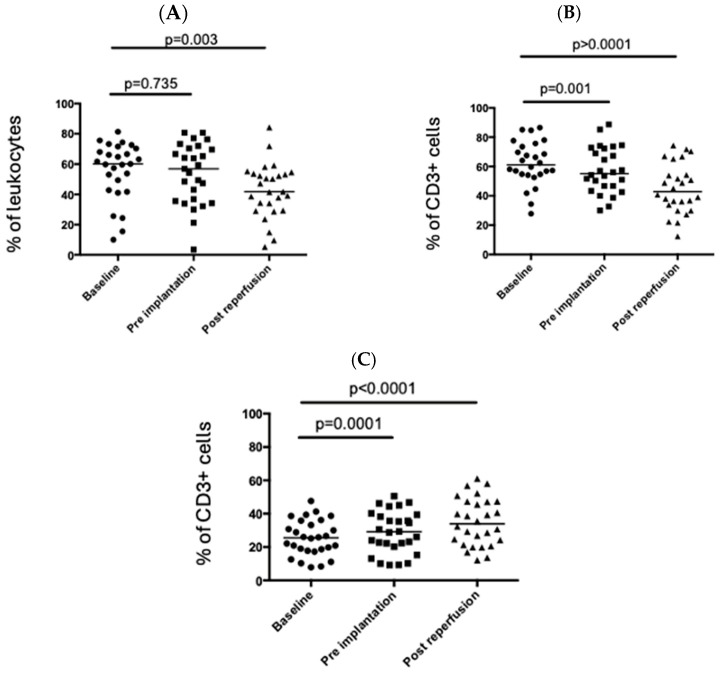
Changes in the T cell populations during the peri-operative period. Immune cells were isolated from the blood at three timepoints during the peri-operative liver transplant period, and T cells were examined by flow cytometry. (**A**) Proportion of CD3+ T cells among lymphocytes. (**B**) Proportion of CD4+ cells. (**C**) Proportion of CD8+ T cells. Each point represents a single patient, and the bar represents the median. Statistical significance was determined by the Friedman test.

**Figure 2:**
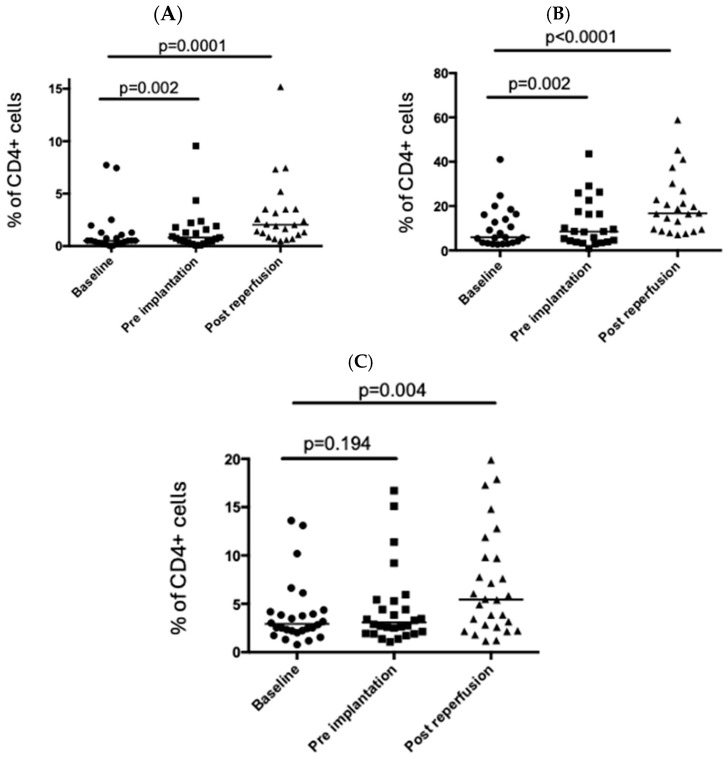
Changes in activation markers on CD4+ T cells in the peri-operative period. Circulating CD4+ T cells were examined at three timepoints in the peri-operative liver transplant period. The proportion of CD4+ T cells expressing CD69 (**A**), CTLA4 (**B**), and HLA-DR (**C**) is shown. Each point represents a single patient, and the bar represents the median. Statistical significance was determined by the Friedman test.

CD3+ T cells (as a percentage of live cells) fell significantly in the peripheral circulation following graft reperfusion (Figure 1A). Further analysis showed that this was driven by a decrease in the percentage of CD4+ T cells in the peripheral circulation with the percentage of CD8+ T cells remaining relatively stable (Figure 1). Intracellular (CTLA4) and cell surface markers (CD69 and HLA-DR; Figure 2A,C) associated with T cell activation were significantly upregulated in peripheral CD4+ T cells post-reperfusion.

### 3.3. Intracellular Production of IFNγ by Circulating CD4+ T Cells Was Significantly Upregulated Following Reperfusion

**Figure 3:**
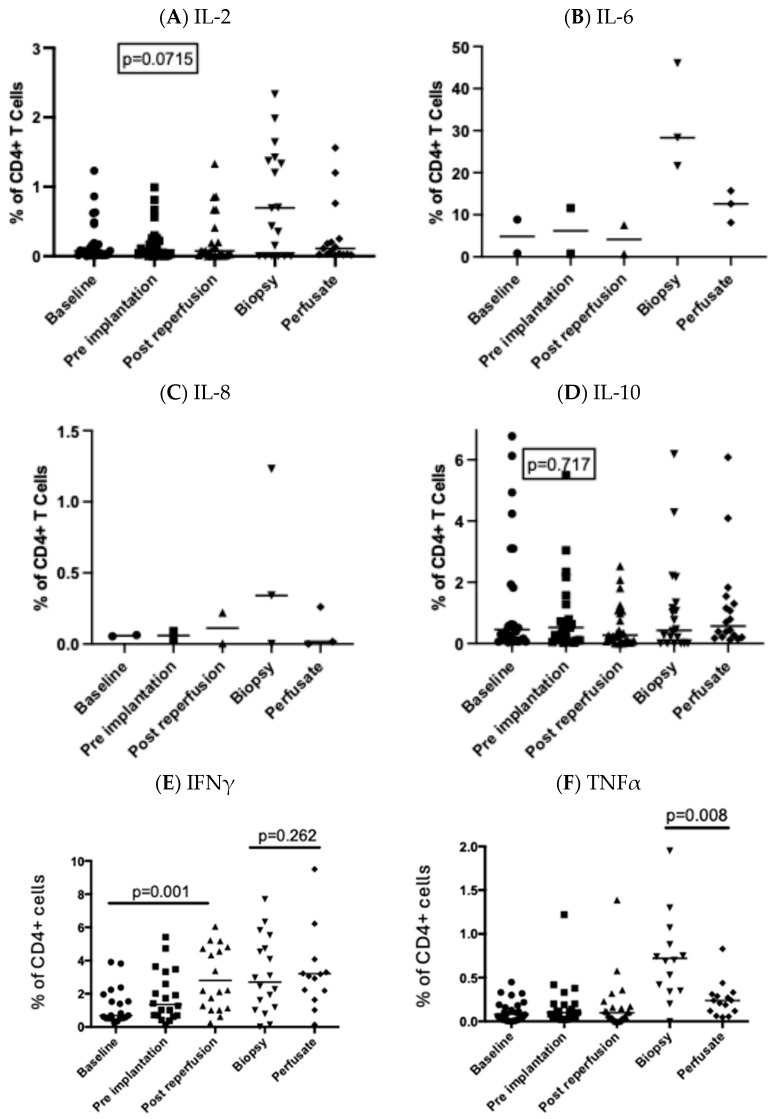
Production of cytokines by CD4+ T cells before and after perfusion of the graft. Circulating CD4+ T cells were examined at 3 timepoints and in the post-reperfusion biopsy and in the transport fluid. The proportion of CD4+ T cells producing IL-2 (**A**), IL-6 (**B**), IL-8 (**C**), IL-10 (**D**), IFNγ (**E**), and TNFα (**F**) is shown. Each point represents an individual patient, and the bar represents the median. Statistical significance was determined by a mixed-effects model.

There was a significantly higher proportion of circulating CD4+ T cells expressing intracellular IFNγ following reperfusion (Figure 3F). This was not reflected in circulating plasma IFNγ levels, which remained unchanged throughout the transplant period (Table 1). The percentage of CD4+ T cells staining positive for TNFα was significantly higher in the tissue compared to the peripheral circulation. CD4+ T cell production of TNFα was significantly upregulated in the liver biopsy compared to intrahepatic CD4+ T cells isolated from the perfusate prior to graft implantation (Figure 3F).

### 3.4. CD69 Was Upregulated on CD8+ T Cells but Their Cytokine Profile Did Not Change Following Reperfusion

There was a significantly higher proportion of circulating CD8+ T cells expressing CD69 following reperfusion (*p* < 0.001). The percentage of CD8+ T cells expressing any measure cytokine (both in the periphery and in the liver) was unchanged across the peri-transplant.

### 3.5. Recipient-Derived Inflammatory Monocytes Are Rapidly Recruited to the Post-Ischaemic Liver

**Figure 4:**
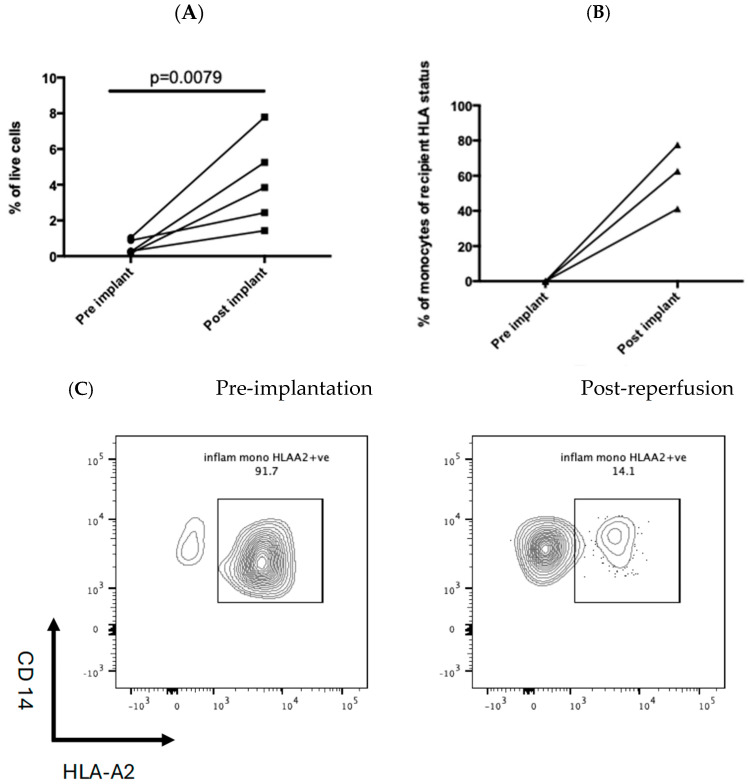
(**A**) Increased number of monocytes in the 5 post-reperfusion biopsies compared to pre-implantation. Each line joins the pre- and post-implant biopsy monocyte number in an individual patient. Statistical significance was determined by the Wilcoxon Signed Ranks test. (**B**,**C**) show an increase in the number of classical monocytes in the post-reperfusion biopsy. The donor was HLA-A2 + ve, and the recipient was HLA-A2-ve.

In five patients, liver biopsies were obtained pre-implantation and post-reperfusion. This demonstrated a significant increase in inflammatory monocytes as a proportion of the total isolated intrahepatic leukocytes following graft reperfusion (Figure 4A). In three donor–recipient pairings, there was a mismatch at the HLA-A2 or HLA-A3 allele. The majority of inflammatory monocytes in the post-reperfusion liver expressed the recipient HLA allele, indicating they were of recipient origin repopulating the graft rapidly (Figure 4B,C).

## 4. Discussion

Liver transplantation is increasingly utilising marginal livers from elderly donors or organs retrieved from cardia death donors, which are prone to liver ischaemia–reperfusion injury. Machine perfusion may have facilitated the use of these more marginal organs [26]. However, the ability to modulate IR injury would make liver transplantation safer and enable the use of more marginal grafts. Understanding the early immune changes following liver transplant would open the door to new opportunities for immune modulation. We present the first study that tracks immune cell changes in the peri-operative period in patients undergoing liver transplantation and one of few studies to monitor early peri-operative cytokine changes. We have shown rapid infiltration of the post-ischaemic liver by recipient-derived inflammatory monocytes and peri-operative cytokine profiles, suggesting innate immune cell activation.

In keeping with our knowledge from murine models, we found that TNFα production by intrahepatic CD4+ T cells predominates in the post-ischaemic liver. The role of CD4+ T cells in murine liver IR has been well documented, and mice lacking CD4+ T cells are protected from liver IR injury [6,7,8]. Furthermore, CD4+ T cells rapidly infiltrate the liver following ischaemic injury [6]. In the present study, there was a reduction in CD4+ T cell numbers in the peripheral circulation shortly after graft implantation, whilst CD8 + T cells were maintained. This drop in circulating CD4+ T cells suggests that they are being recruited to areas of inflammation such as the implanted graft. Unfortunately, graft uptake of the circulating CD4+ T cells could be inferred but not proven as the pre-implant liver graft biopsy only yielded sufficient material for one panel, and we chose to prioritise monocyte recruitment.

Early markers of peripheral CD4+ T cell activation were also upregulated during hepatic mobilisation and following reperfusion of the liver. Taken together, these findings align with findings from small-animal models, in that CD4+ T cells are rapidly recruited to the post-ischaemic liver [6].

Interestingly, inflammatory cytokines were seen to rise intra-operatively and were elevated when measured immediately prior to recipient hepatectomy and before graft implantation. This likely reflects that significant tissue injury has occurred, and we know from previous studies that liver ischaemia–reperfusion injury can occur during liver mobilisation even in the absence of inflow occlusion [27] [Liver manipulation causes hepatocyte injury and precedes systemic inflammation in patients undergoing liver resection. Van de Poll et al. World Journal of Surgery 2007 31, 10 2033–8]

The most striking upregulation of cytokine production following transplant by circulating CD4+ T cells was that of IFNγ. IFNγ produced by CD4+ T cells plays a key role in driving M1 macrophage response [28] and is a potent activator of monocytes [29]. In a study investigating renal IR injury in a mouse model, adoptive transfer of CD4+ T cells from IFNγ-deficient mice into athymic mice did not result in IR injury, suggesting that renal IR is related to IFNγ rather than other functions of CD4+ T cells [9]. However, the evidence is conflicting as IFNγ blockade in a murine model of hepatic IR injury did not protect against IR injury, whilst CD40L blockade did [13]. Small-animal models have demonstrated a key role for CD40–CD40L interactions. CD40L blockade or depletion has been shown to ameliorate IR injury in the mouse [30] and rat [31] liver. This confirms an interplay between CD4+ T cells and the innate immune response as a key mechanism of hepatic IR injury and is in keeping with our findings showing the upregulation of IFNγ production by CD4+ T cells.

The key finding of this study was the rapid infiltration of inflammatory monocytes into the post-ischaemic liver. Three donor–recipient pairs had a mismatch of an HLA- A2 or HLA-A3 allele. This allowed clear demonstration that inflammatory monocytes present in the liver shortly after reperfusion were derived from the recipient and not the donor. This would suggest that strategies for modulating the inflammatory monocyte response should therefore be directed at the recipient and not the donor. There is emerging evidence from murine studies that inflammatory monocytes play a key role in hepatic IR injury [14] and are recruited to the liver via CCR2. This is in keeping with our results, which have shown increased circulating levels of CCL2 and that CCL2 levels measured at 24 h correlated with levels of the marker of acute liver injury, AST levels at day 3. AST on day 3 has been shown to be a surrogate marker of patient and graft outcomes following liver transplant [25] and may reflect ongoing damage to the graft. Interestingly, patients who experienced a graft-related complication including those with early allograft dysfunction [24] had higher circulating levels of IL-10 post graft reperfusion. Monocytes that are activated via TLR4 have been shown to secrete high levels of IL-10 [32], and this may therefore reflect innate immune cell activation and damage. Monocytes have been shown to display plasticity in their response to sterile injury in the liver, and over time, inflammatory monocytes change their phenotype to promote repair [16]. Further studies are required to gain a deeper understanding of the phenotyping of the monocytes recruited to the post-ischaemic liver as phenotypical manipulation of this cell population presents an attractive putative target for ameliorating liver IR injury. Furthermore, an understanding of the mechanisms by which monocytes interact with CD4+ T cells and drive cellular injury following liver IR injury will give a greater understanding of this complex pathway.

The incidence of EAD in this population (54%) is higher than in the published literature, which ranges from 20 to 40% [33]. Although EAD has been associated with increased graft loss and poorer long-term graft function, this is not reflected in our results showing excellent graft and patient outcomes. This may be due to 10 patients being classified as EAD due to high transaminases in the immediate post-operative period that reduced quickly by day 1 post-op. This is reflected in our own analysis of the role of transaminases in the immediate post-operative period and correlations with graft and patient outcomes [25], which found that high transaminases on day 3 were a better predictor of graft survival as it likely demonstrated ongoing rather than immediate graft injury.

This study has several limitations. As it examined immune cell and cytokine profiles in human patients undergoing liver transplantation, it is observational in nature and sample timepoints were limited. However, this is also a strength of this study. There are significant differences between the immune response in mice and humans [34], and to our knowledge, this is the first study to track immune cells in humans in the peri-transplant period. It has confirmed some similarities to rodent models of IR injury, including the infiltration of inflammatory monocytes, the upregulation of CCL2, and evidence of major shifts in the CD4+ T cell population. The initial aim of this study was to track CD4+ T cells, and therefore, although there were interesting findings regarding the recruitment of inflammatory monocytes, we were not able to analyse this specific cell population for cytokine production and markers of activation. Further studies will be required to interrogate this in more detail.

In conclusion, the results from this study have shown the extravasation of CD4+ T cells and their activation in liver transplantation. We have shown the recruitment of recipient inflammatory monocytes to the human liver following transplantation, and this presents an interesting option for early modulation of the immune response.

## Figures and Tables

**Table 1:** Baseline characteristics.

Donor	
Sex (M/F)	21/7
Age	50 (18–59)
DBD/DCD	32/5
DRI	1.61 (1.42–2.03)
**Recipient**	
Sex (M/F)	24/4
Age	56 (49–64)
MELD	15 (11–16)
UKELD	54 (50–59)
**Transplant**	
Cold ischaemic time (min)	478 (393–617)
Anastomosis time (min)	43 (37–50)
Operative time (min)	453(389–504)
RCC transfusion (units)	3 (1–5)
Piggyback/caval replacement	14/14
Peak AST (iU/L)	2190 (721–3204)
Peak ALT (iU/L)	1513 (672–2624)
Day 3 AST (iU/L)	302 (103–443)
EAD	15

Abv: DCD—donor following cardiac death, DRI—donor risk index, MELD—model for end-stage liver disease, UKELD—United Kingdom model for end-stage liver disease, RCC—red cell concentrate, AST—aspartate transferase, ALT—alanine transferase, EAD—early allograft dysfunction.

**Table 2:** Plasma cytokine levels during the transplant period (median + IQR) * denotes significance identified with the Friedman test.

Cytokine	Baseline (pg/mL)	Pre-Implantation (pg/mL)	Post-Reperfusion (pg/mL)	24 h Post-Op (pg/mL)
CCL2 *	69.13 (53.96–104.1)	154.89 (118.54–211.63)	384 (205–765.12)	74.66 (43.98–138.44)
CCL5 *	2288.82 (1246.91–3786.05)	4745.69 (2916.86–8015.93)	3209.15 (1741.91–5843.55)	2737.84 (1668.26–3209.49)
IL-2	9.34 (4.08–35.11)	7.50 (4.08–40.40)	6.19 (4.08–17.13)	12.16 (4.08–42.29)
IL-6 *	13.98 (8.27–44.80)	245 (150.97–375.12)	644.98 (338.31–1132.01)	21.58 (10.11–43.29)
IL-8 *	0.88 (0–3.26)	8.23 (1.28–15.84)	30.59 (15.37–52.42)	0.88 (0–3.11)
IL-10 *	4.22 (3.72–7.69)	9.83 (4.38–16.96)	540.74 (344.21–815.48)	7.37 (4.56–35.26)
IL-17A *	2.14 (1.74–2.96)	2.40 (1.68–3.2)	2.94 (1.85–8.78)	1.86 (0.81–2.33)
IFNγ	57.13 (18.905–176.09)	32.66 (17.15–73.29)	31.41 (10.92–107.48)	17.35 (6.50–44.20)
TNFα	7.97 (3.5–53.16)	7.17 (3.5–33.79)	7.15 (5.46–8.58)	6.46 (3.5–8.58)

Abv: CCL2—Chemokine Ligand 2, CCL5—Chemokine Ligand 5, IL-2—Interleukin 2, Il-6—Interleukin 6, IL-8—Interleukin 8, IL-10—Interleukin 10, IL-17A—Interleukin 17A, IFNγ—Interferon Gamma, TNFα—Tumour Necrosis Factor Alpha.

## Data Availability

Further data can be made available by contacting the corresponding author.

## Supplementary Materials

The following supporting information can be downloaded at https://www.mdpi.com/article/10.3390/cimb47010049/s1.
