# Supplementary material for: Inflammatory Monocytes Are Rapidly Recruited to the Post-Ischaemic Liver in Patients Undergoing Liver Transplantation and Cytokines Associated with Their Activation Correlate with Graft Outcomes
Source: Curr Issues Mol Biol. 2025 Jan 14;47(1):49. doi: 10.3390/cimb47010049 (PMC11763458; doi:10.3390/cimb47010049)
Supplement: Supplementary file 1 [file cimb-47-00049-s001.zip › cimb-3366132-supplementary.pdf]

Supplementary file:

1: Sample collection and processing

Blood

Twenty-five mls of peripheral arterial blood was collected at 4 time-points during the transplant period (under Methods). Twenty mls was collected in BD Vacutainer EDTA blood tubes (BD, UK) and five mls was collected in BD Vacutainer plasma blood tubes containing heparin (BD, UK).

Liver biopsies

A tru-cut liver parenchymal biopsy was taken pre implantation and at 2 hours post portal vein reperfusion. The biopsy was immediately placed in RPMI 1640 media without L glutamine (Lonza, UK).

Processing of whole blood

20mls of whole blood was diluted with 15mls of Phosphate Buffered Saline (PBS) and 13mls of Ficoll was layered under the diluted blood. The sample was centrifuged (940g, 20 minutes, 20^0C). The peripheral blood mononuclear cells (PBMCs) were then removed and counted. 5 million cells were reconstituted in PBS for analysis and excess cells were frozen, in aliquots of 5 million cells, in 1ml of heat inactivated FCS with 10% DMSO (Sigma, UK) for later analysis.

Plasma was obtained by centrifuging the plasma tube (10 mins, 300g, 20^0C). The plasma was then aliquoted into 750µl samples and stored at -80^0C till further analysis.

Processing of liver biopsy

The biopsy was divided into smaller segments with a cell scraper and was passed through a 40µm filter into 30mls of RPMI 1640 media without L glutamine (Lonza, UK) to obtain the intrahepatic lymphocytes (IHLs). The solution was centrifuged (500g, 5 mins, 4^0C), Cells were cultured in 1ml T cell media with 5µl Brefeldin A (amounting to a 10µg/ml concentration) added (4 hours, 5% CO2, 37^0C). The cells were centrifuged (500g, 5 mins, 4^0C) and resuspended in 1ml of PBS ready for staining for flow-cytometry.

Antigen	Conjugate	Source	Clone	Dilution	RRID
Live/Dead	Near IR (780)	Life Technologies (#L34992)	n/a	(1:1000)	NA
Extracellular					
CD3	PE-CF594	BD (#562310)	UCHT1	(1:200)	AB_2646222
CD4	PE Cy7	eBioscience (#25-0049-41)	RPA-T4	(1:100)	AB_1659697
CD8a	Alexa Fluor 700	eBioscience (#56-0086-82)	OKT-8	(1:200)	AB_657756
HLADR	V500	BD (#561224)	G46-6	(1:100)	AB_10563765
CD69	FITC	BD (#560969)	FN50	(3:100)	AB_10562195
PD-1	PerCP Cy5.5	Biolegend (#329913)	EH12.2H7	(1:50)	AB_1595561
Intracellular					
CTLA-4	PE	BD (#557301)	BN13	(3:100)	AB_396628
FoxP3	Pacific Blue	Biolegend (#320215)	259D	(1:25)	AB_940354
IL-2	PerCP eFlour 710	eBioscience (#46-7029-42)	MQ1-17H12	(1:50)	AB_1834419
IL-10	PE	eBioscience (#12-7108-82)	JES3-9D7	(1:100)	AB_466179
IFNγ	FITC	R&D systems (#IC285F-100)	♯25723	(3:100)	AB_357308
pS6	Alexa Fluor 647	Cell Signalling (#4851S)	D57.2.2E	(1:100)	AB_10695457
TNFα	APC	Biolegend (#502913)	MAb11	(1:400)	AB_315265

Supplementary table S1: antibodies used to analyse T cell populations.

Antigen	Conjugate	Source	Clone	Dilution	RRID
Live/Dead	eFluor450	eBioscience (#65-0863-18)	n/a	(1:1000)	NA
Extracellular					
CD3	APC eFluor 780	eBioscience (#47-0036-42)	SK7	(1:200)	AB_10717514
CD4	PE eFlour 610	eBioscience (#61-0049-42)	RPA-T4	(1:200)	AB_2574522
CD8	PE	BD (#555367)	RPA-T8	(1:200)	AB_395770
CD14	PerCP	Invitrogen (#MHCD1431)	TUK4	(1:200)	AB_10374157
CD16	PE	BD (#555407)	3G8	(1:200)	AB_395807
CD19	APC eFluor 780	eBioscience (#47-0199-42)	HIB1.9	(1:100)	AB_1582230
CD56	BV510	BD (#563041)	NCAM16.2	(1:200)	AB_2732786
HLA-A2	APC	eBioscience (#17-9876-42)	BB7.2	(1:100)	AB_11149299
HLA-A3	FITC	eBioscience (#11-5754-42)	GAP.A3	(1:50)	AB_2572503

Supplementary table 2: Antibodies used to analyse monocyte populations.

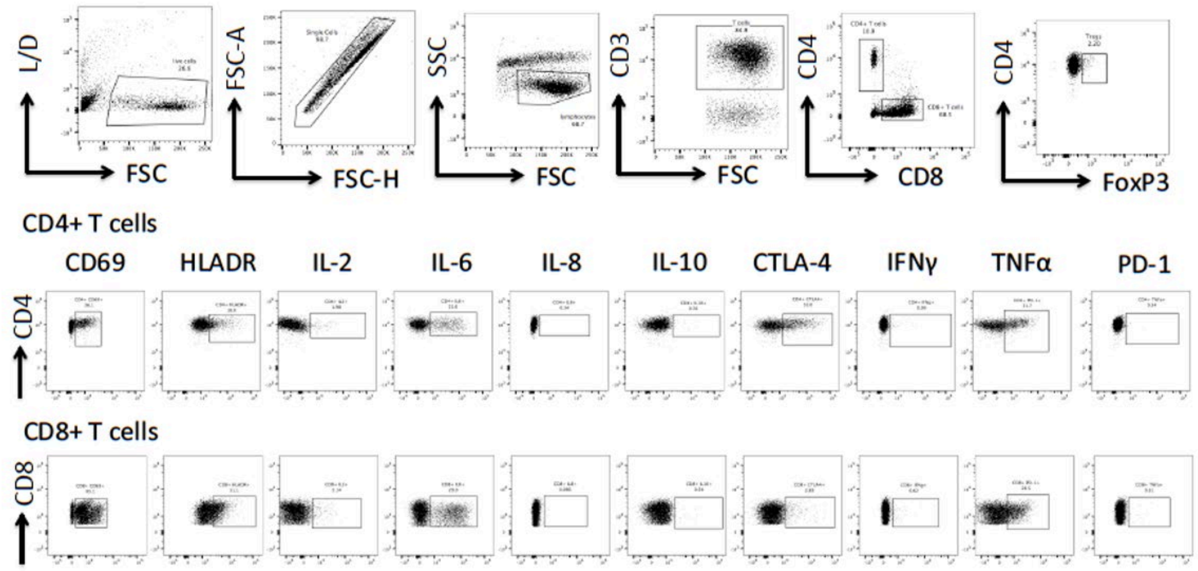

Supplementary figure 1: Gating strategy for CD4+ and CD8+ T cells.

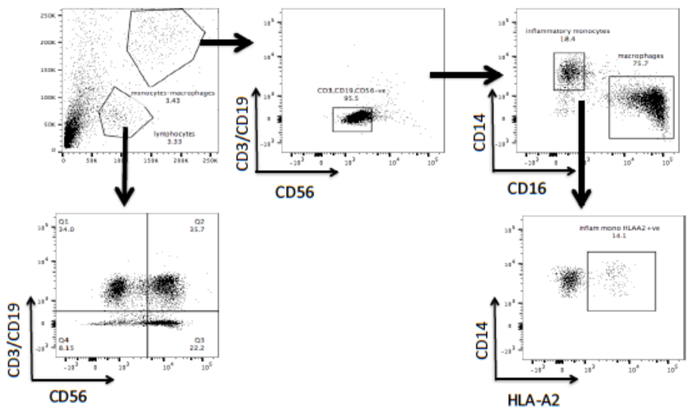

Supplementary figure 2: Gating strategy for inflammatory monocytes
